# Venous thromboembolism: A problem in the Indian/Asian population?

**DOI:** 10.4103/0970-1591.45531

**Published:** 2009

**Authors:** Sunil Agarwal, Arvind Dhas Lee, Ravish Sanghi Raju, Edwin Stephen

**Affiliations:** Department of General Surgery Unit II and Vascular Surgery, Christian Medical College, Vellore-632 004, Tamil Nadu, India; 1 Vascular Surgery, Adelaide University, Adelaide, Australia

**Keywords:** Thromboembolism, urology, venous

## Abstract

Venous thromboembolism (VTE) is a common and potentially life threatening condition. It continues to be under diagnosed and undertreated. Awareness among Indians regarding this potentially life-threatening disease is low. Contrary to earlier belief, the incidence of VTE in Asia and India is comparable to that in Western countries. The risk of VTE is especially high in hospitalized patients, in a majority of whom it is clinically silent. It is one of the commonest causes of unplanned readmission and preventable death. In the United States, it is responsible for more deaths than accidents. Thromboprophylaxis is highly effective in reducing the incidence of VTE without any increase in clinically significant bleeding. It is worth emphasizing that prevention of VTE is much easier and cheaper than its treatment.

## INTRODUCTION

Quoted as a major health problem and one of the most common preventable causes of hospital deaths in the western world, venous thromboembolism (VTE) has rarely evoked such consideration in India. Not surprisingly, a Pubmed search of “Deep vein thrombosis + India” yields just 8 articles. Deep venous thrombosis (DVT) and pulmonary embolism (PE) are a single clinico-pathological entity - venous thromboembolism (VTE). The true incidence of VTE is hard to get because of the often silent nature of the condition. In the western world, the incidence is one case of DVT and 0.5 cases of PE per 1000 population/year.[1] Hospitalized patients are especially at risk for VTE as most have multiple risk factors. Autopsy studies have shown the incidence of VTE in hospitalized patients to be as high as 34.7% with fatal pulmonary embolism in 9.4%. Table 1 shows the absolute risk of VTE in hospitalized patients.[2,3]

**Table 1:** Absolute risk of venous thromboembolism in hospitalized patients

Patient group	VTE prevalence (%)
Medical patients	10-20
Cardiac patients	15-40
Major gynaecological surgery	15-40
Major urological surgery	15-40
Neurosurgery	15-40
Stroke	20-30
Hip and knee arthroplasty	40-60
Major trauma	40-50
Spinal cord injury	60-80
Critical care patients	10-20

In this article, we give an overview of the epidemiology, pathophysiology, treatment, and prevention of VTE and its complications. Special emphasis is laid on recent studies from the Indian/Asian population. We also present data from studies done on urological patients to assess the risk of VTE and to make recommendations regarding VTE prophylaxis.

Most studies from India have looked at specific patient groups like postoperative orthopaedic patients[4,5] and there is no data on the overall incidence of VTE in the general population. The prevailing belief that VTE in the Asian population is less than in the Western population has essentially been disproved[6–9] and there appears no reason to believe that it should be any different in India.

## PATHOPHYSIOLOGY AND RISK FACTORS

Virchow's far-sighted observation on the triad of factors leading to venous thrombosis has, with some refinement, stood the test of time. The triad of change in blood flow, change in blood constituents, and change in vessel wall has now been refined to venous stasis, activated coagulation pathways, and venous endothelial injury.

Venous thrombi are made up of fibrin, red cells, platelets, and leucocytes. Typically, these thrombi are believed to start in areas of slow or turbulent venous flow such as large venous sinuses or venous valve cusps and also in areas of direct venous trauma. Activation of the coagulation pathway is the crucial step in the initial formation of venous thrombi and this is believed to happen due to local injury or remote release of mediators. Activation of the pathway alone is inadequate in formation of a full-fledged venous thrombus as inhibitors of thrombosis such as antithrombin and thrombomodulin-protein C and S, tissue factor pathway inhibitor (TFPI) along with the fibrinolytic pathway would clear the clot. Hence, it is persistent activation due to endothelial stimulation along with poor flow failing to clear the activated factors that results in an imbalance in the pro and anti-thrombotic pathways which ultimately leads to progression of the thrombus.

Heit and colleagues have listed the following conditions as major risk factors for developing VTE: increasing age, male gender, surgery, trauma, confinement in hospitals or nursing homes, malignancy, neurologic disease, central venous catheter, prior superficial vein thrombosis, and varicose veins.[10] Pregnancy, oral contraceptive pill use, and hormone replacement therapy are independent risk factors in women.

The surgical patient has all three Virchow's factors present in the peri-operative period. They have venous stasis due to immobilization and surgical positioning. Direct venous injury or remote release of mediators of coagulation due to tissue trauma also increases the risk of venous thrombosis. The risk factors for a surgical patient developing VTE have been extensively studied and the important determinants appear to be age, type of surgery, length of procedure, and duration of immobilization. Hull *et al*.[11] have categorized post operative patients into low, moderate and high risk for VTE on the basis of these characteristics [Table 2].

**Table 2:** Stratification of patients based on their risk for developing venous thromboembolism

Category	Characteristics
Low	Age <40 years, no other risk factors, uncomplicated abdominal/thoracic surgery
	Age >40 years, no other risk factors, minor elective abdominal/thoracic surgery <30 min
Moderate	Age >40 years, abdominal/thoracic surgery >30 min
	History of recent thromboembolism
High	Abdominal or pelvic procedure for malignancy
	Major lower extremity orthopaedic procedure

## NATURAL HISTORY

Acute DVT is followed by a complex process of attempted recanalization of the vessel lumen which is mediated by leukocyte infiltration and cell mediated thrombolysis. In animal models, the recanalization process has been found to begin within a few days of the initial thrombosis and complete recanalization of the vessel lumen is the most common outcome.

Rethrombosis would naturally impede with the recanalization process and recurrent thromboembolism of up to 47% has been reported in patients inadequately anti-coagulated in the first 3 months after an initial proximal DVT.[12] The clinical behavior of acute DVT depends on the location of thrombosis. In the lower limb, proximal iliofemoral DVTs tend to cause more acute and chronic complications than distal calf vein DVTs. Calf vein DVTs tend to recanalize faster than proximal ones.

Pulmonary embolism is the most dangerous complication of acute DVT. As with acute DVT, this usually remains clinically silent. 25-50% of all patients with documented DVT and absence of pulmonary symptoms have been shown to have evidence of PE on lung perfusion scans.[13] Symptomatic pulmonary embolism is strongly associated with inadequately treated DVT and underlying cardio-pulmonary reserve of the patient. The mortality rate of PE is 11% within an hour of presentation and a further 30% among survivors if not recognized.[14]

## DIAGNOSIS

The clinical diagnosis of DVT is generally inaccurate, especially in the inpatient setting. Of patients undergoing ultrasound “to rule out DVT”, only about 15% are found to have one.[15] To better select patients for screening, Wells *et al*.[16] have suggested the following clinical model to assess pre-test probability of DVT [Table 3].

**Table 3:** Clinical model to assess pre-test probability of deep vein thrombosis

Clinical feature	Score
Active cancer (treatment ongoing or within previous 6 months or palliative)	1
Paralysis, paresis, or recent plaster immobilization of the legs	1
Recently bedridden for more than 3 days or major surgery within 4 weeks	1
Localized tenderness along the distribution of the deep venous system	1
Entire leg swollen	1
Calf swelling by more than 3 cm compared with the asymptomatic leg (measured 10 cm below the tibial tuberosity)	1
Pitting oedema (greater in the symptomatic leg)	1
Collateral superficial veins (non-varicose)	1
Alternative diagnosis as likely or wider than that of deep vein thrombosis	-2

Low probability: score of 0 or less; Moderate probability: 1-2; High probability: 3 or more.

The value of a clinical probability score along with D-dimer assessment is useful to eliminate the possibility of DVT. Plasma D-dimers are derivatives of fibrin degradation products and are found to be raised in patients with thromboembolism. Plasma D-dimer assay has very high sensitivity but poor specificity for venous thrombosis and hence a patient with low probability of DVT with a negative D-dimer test has almost no chance of having venous thrombosis.

Duplex ultrasonography has replaced venography as the investigation of choice in diagnosing DVT. It is cheap, easily available, and non-invasive. However, it does have some disadvantages that need to be kept in mind - diagnosis of isolated calf vein DVT and evaluation of iliac veins is often dependent on operator experience, patient habitus, and the clinical situation. Despite these limitations, a negative report from a single, technically adequate ultrasound examination is sufficient grounds to withhold anticoagulation therapy. A complete ultrasound performed by trained sonologists with a proper protocol, resulted in only 4 (1.1%) instances of misdiagnosis in more than 400 patients.[17] Computerized tomography (CT) pulmonary angiography is the most useful test for detecting a pulmonary embolus. It can be combined with CT venography as a single scan to diagnose both PE and DVT.

## TREATMENT

Once the diagnosis of DVT has been established, treatment involves anticoagulation to encourage clot lysis and recanalization and prevent re-thrombosis and embolization. Traditionally, this involved a continuous infusion of unfractionated heparin followed by oral anticoagulation with warfarin for a period of 3-6 months. Unfractionated heparin consists of a heterogeneous mixture of polysaccharide chains and hence, its therapeutic action can be extremely variable. This necessitates the need to monitor therapy using either activated partial thromboplastin time (aPTT) or heparin blood levels and titrating the dose based on these.

Low molecular weight heparins (LMWH) are derivatives of unfractionated heparin formed by depolymerization. The advantage they have over unfractionated heparin is that the anticoagulant response to a standard dose of LMWH is consistent and predictable and hence does not require monitoring during therapy. Also, the half life of these drugs is longer and once-daily dosage is possible. Several trials have shown that LMWH are at least as effective as unfractionated heparin in the treatment of DVT[18–21] and the risk of bleeding related complications appears to be less.[22] Continued anticoagulation is usually maintained using oral warfarin.

The major complication of anticoagulant therapy is bleeding. Heparin can also result in an immune mediated thrombocytopenia called Heparin Induced thrombocytopenia (HIT) syndrome which can result in wide spread arterial and venous thrombosis. If HIT syndrome is diagnosed then heparin in all forms has to be stopped and anticoagulation using other agents such as hirudin and argatroban will have to be instituted.

The Sixth ACCP Consensus Conference on Antithrombotic Therapy has made the following recommendations for duration of anticoagulation: patients with reversible or temporary risk factors for VTE may be treated for 3-6 months, while patients with irreversible risk factors such as thrombophilic states and malignancy are to be treated indefinitely.[23]

## PROPHYLAXIS

There are two ways in which pulmonary embolism, the most serious complication of DVT can be prevented - primary prevention of the initial DVT and secondary prevention by early detection and treatment of DVT. Given the poor sensitivity of clinical diagnosis of DVT, primary prevention has become the preferred mode of prophylaxis. Methods of primary prevention include early and persistent mobilization, pharmacological agents like unfractionated heparin and low molecular weight heparin (LMWH) and mechanical means like intermittent pneumatic compression and graduated compression stockings.

Patients at low risk should be encouraged early mobilization and do not require prophylaxis. Patients with moderate risk should have additional pharmacological prophylaxis. Patients in the high-risk group require pharmacological and mechanical prophylaxis. In patients undergoing surgery in which minor haemorrhage may be critical to the outcome, such as neurosurgery, ophthalmic surgery, and spine surgery, mechanical prophylaxis is recommended.

In surgical patients, the prothrombotic state begins intraoperatively and hence prophylaxis should ideally commence before induction of anaesthesia. Trials have shown that initiating prophylactic anticoagulation 2 h prior to surgery is both safe and effective in decreasing the risk of fatal PE.[2,24] If prophylaxis cannot be started peri-operatively because of the risk of bleeding, there is still a beneficial effect in starting it postoperatively. Prophylaxis in the postoperative period is generally maintained till the patient is discharged. However, in high-risk orthopaedic procedures such as hip replacements, extended prophylaxis up to 30 days is indicated.

Prophylactic anticoagulation in the setting of regional neuraxial anaesthesia poses the risk of a spinal hematoma which can potentially lead to permanent neurological impairment. Specific risk factors for this happening are prolonged epidural analgesia, traumatic introduction, and females over the age of 75 years. Specific guidelines on the usage of prophylactic anticoagulation, in this setting, have been outlined by the American Association of Regional Anesthesia Consensus Conference.[25]

Among the various pharmacological agents, meta-analyses have shown that LMWH and unfractionated heparin have equal efficacy in preventing VTE in general surgical patients. However, the group on unfractionated heparin had a higher incidence of minor bleeding episodes such as wound hematomas.[26,27] Intermittent pneumatic compression alone is a useful method of thromboprophylaxis in general surgical and urological patients at high risk of bleeding. However, it is limited by patient compliance and ease of use. Graduated compression stockings are simple to use and moderately effective. They have been shown to increase the velocity of venous blood flow. They are recommended in patients at low risk for VTE and can be combined with pharmacological prophylaxis in patients at medium to high risk. Its usage is contraindicated in patients with peripheral vascular disease.

## PULMONARY EMBOLISM

Fatal PE is the most dramatic effect of VTE and remains the most common preventable cause of death in hospitalized patients. The best strategy is prevention by adequate prophylaxis for VTE. Early detection is essential for improved out come and requires a high degree of suspicion, when patients have:

- Unexplained tachycardia
- Unexplained fever
- Chest pain
- Dyspnea
- Hemoptysis

Treatment of PE is decided by the cardiovascular status of the patient. If the patient has circulatory collapse then he is best managed by catheter directed thrombolysis or thrombectomy. If the patient is stable than adequate anticoagulation is sufficient.

**Figure 1:**
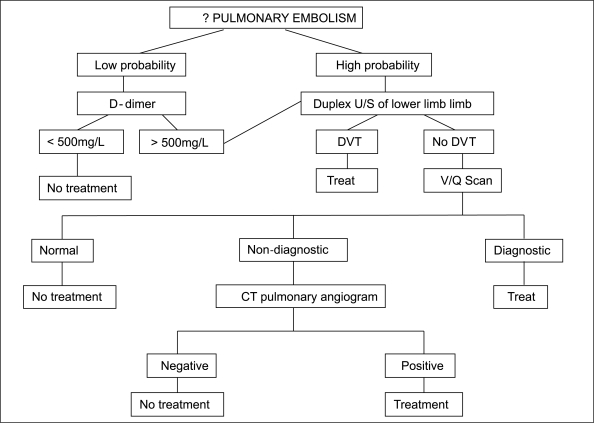
Diagnostic strategy for the investigation of suspected pulmonary embolism

## THE UROLOGICAL PATIENT

DVT is considered to be the most important nonsurgical complication following major urological procedures while PE is believed to be the most common cause of postoperative death.[28] Patients undergoing major urological surgery often have multiple risk factors for VTE like advanced age, malignancy, use of the lithotomy position, and pelvic surgery with or without lymph node dissection. Despite this, the role of thromboprophylaxis in urological surgery has not received much attention. A recent prospective observational study compared the incidence of VTE in patients undergoing surgery for urological cancers with general and gynaecological cancers. It suggests that while there is still significant risk in urological patients, the risk is less than general or gynaecological patients.[29] A survey of current urological practice in patients undergoing radical prostatectomy at various centers in 3 countries showed widely disparate usage of thromboprophylaxis.[30] Evidence from colorectal and gynaecological surgery would suggest that the risk of VTE in major pelvic urological surgery ought to be significant and prophylaxis with LMWH or low dose unfractionated heparin in conjunction with compression stockings is probably indicated. A retrospective analysis on the incidence of VTE in patients undergoing transurethral resection of the prostate (TURP) with limited prophylaxis, concluded that these patients are at low risk for clinically evident DVT and at intermediate risk of clinically evident PE.[31] This study seems to suggest that some form of pharmacological prophylaxis is essential to prevent PE in patients undergoing TURP. Obviously, further studies are required for making recommendations on the use of thromboprophylaxis in endo-urological procedures.

The current recommendation for VTE prophylaxis in Urological surgery is as follows:[32]

1. For patients undergoing transurethral or other low-risk urologic procedures, recommendation against the use of specific thromboprophylaxis other than early and frequent ambulation.
2. For all patients undergoing major, open urologic procedures, thromboprophylaxis be used routinely.
3. For patients undergoing major, open urologic procedures, routine thromboprophylaxis with low dose unfractionated heparin twice daily or three times daily, graduated compression stockings and/or intermittent pneumatic compression started just before surgery and used continuously while the patient is not ambulating, low molecular weight heparin, fondaparinux, or the combination of a pharmacologic method with the optimal use of a mechanical method.
4. For urologic surgery patients who are actively bleeding or who are at very high risk for bleeding, optimal use of mechanical thromboprophylaxis with graduated compression stockings and/or intermittent pneumatic compression at least until the bleeding risk decreases. When the high bleeding risk decreases, pharmacologic thromboprophylaxis be substituted for or added to the mechanical thromboprophylaxis.
